# Predicting multi-level drug response with gene expression profile in multiple myeloma using hierarchical ordinal regression

**DOI:** 10.1186/s12885-018-4483-6

**Published:** 2018-05-10

**Authors:** Xinyan Zhang, Bingzong Li, Huiying Han, Sha Song, Hongxia Xu, Yating Hong, Nengjun Yi, Wenzhuo Zhuang

**Affiliations:** 1 Department of Biostatistics, Jiann-Ping Hsu College of Public Health, Georgia Southern University, Statesboro, GA USA; 2 Department of Hematology, The Second Affiliated Hospital of Soochow University, Suzhou, China; 3 Department of Cell Biology, School of Biology & Basic Medical Sciences, Soochow University, Suzhou, China; 4 Department of Biostatistics, University of Alabama at Birmingham, Birmingham, AL 35294 USA

**Keywords:** Gene expression, Hierarchical ordinal regression, Multiple myeloma, Multi-level drug response, Prediction

## Abstract

**Background:**

Multiple myeloma (MM), like other cancers, is caused by the accumulation of genetic abnormalities. Heterogeneity exists in the patients’ response to treatments, for example, bortezomib. This urges efforts to identify biomarkers from numerous molecular features and build predictive models for identifying patients that can benefit from a certain treatment scheme. However, previous studies treated the multi-level ordinal drug response as a binary response where only responsive and non-responsive groups are considered.

**Methods:**

It is desirable to directly analyze the multi-level drug response, rather than combining the response to two groups. In this study, we present a novel method to identify significantly associated biomarkers and then develop ordinal genomic classifier using the hierarchical ordinal logistic model. The proposed hierarchical ordinal logistic model employs the heavy-tailed Cauchy prior on the coefficients and is fitted by an efficient quasi-Newton algorithm.

**Results:**

We apply our hierarchical ordinal regression approach to analyze two publicly available datasets for MM with five-level drug response and numerous gene expression measures. Our results show that our method is able to identify genes associated with the multi-level drug response and to generate powerful predictive models for predicting the multi-level response.

**Conclusions:**

The proposed method allows us to jointly fit numerous correlated predictors and thus build efficient models for predicting the multi-level drug response. The predictive model for the multi-level drug response can be more informative than the previous approaches. Thus, the proposed approach provides a powerful tool for predicting multi-level drug response and has important impact on cancer studies.

**Electronic supplementary material:**

The online version of this article (10.1186/s12885-018-4483-6) contains supplementary material, which is available to authorized users.

## Background

Multiple myeloma (MM) is a malignant plasma cell disorder characterized by the proliferation in the bone marrow of clonal plasma cells [1, 2]. Around 30,280 new multiple myeloma cases are expected to be diagnosed in 2017 [3]. Meanwhile, MM is an incurable disease using conventional treatment, which results in a median overall survival of 3 to 4 years [4, 5]. Nevertheless, treatment outcome in MM has improved significantly in the last decade, partially due to the introduction of novel agents, such as the proteasome inhibitors (e.g. bortezomib) and immunomodulatory drugs (e.g. thalidomide) [6]. In spite of that, the heterogeneity exists in the patients’ response to those new treatments and molecular features responsible for the variability in response remain undefined [7–9]. It urges more efforts to identify biomarkers from numerous molecular features and build predictive models for identifying patients that can benefit from a certain treatment scheme [7].

MM, like other cancers, is caused by the accumulation of genetic abnormalities [2, 10]. Various molecular analyses suggest that myeloma is composed of distinct subtypes that have different molecular pathologies and prognosis [10]. For example, cytogenetic studies reveal that 60 to 80% of myeloma cases reveal chromosomal translocations involving the immunoglobulin heavy (IgH) locus [10]. The most prevalent of these translocations are t(11;14)(q13;q32) and t(4;14)(p16;q32), where the former has better survival than the latter. Another chromosomal translocation in t(8;14)(q24;q32), causing MYC activation, is considered as a secondary hit. Other genetic abnormalities include mutations and copy number changes. Mutational spectrum reveals a heterogeneous landscape with few recurrently affected genes. Only three genes have been reported in more than 10% patients, including *KRAS*, *NRAS*, and *FAM46C* [11–14]. For copy number changes, the most common ones being gains are on 1q, 3p, 6p, 9p, 11q, 19p, 19q and 21q along with deletions of 1p, 4q, 16q and 22q, among which candidate oncogenes and tumor suppressors have been identified [15–18]. Thus, it is anticipated that identifying and applying molecular biomarkers to predict clinical response to drugs will help to provide more precise prognostic and predictive classifiers for a specific therapy in MM.

With the emergence of high-throughput sequencing technology, it is expected that the number of biomarkers will rise. The markers in predicting drug response will become more reproducible as well [19, 20]. However, the effect sizes of the discovered markers are usually small, which only could contribute a relatively trivial portion to the drug response since it is typically a complex trait, generally influenced by many genomic and environmental factors [19]. Thus, predictive modeling with multiple markers should be used to predict complex traits, such as drug response [19].

Distinct gene expression profiling is believed to be associated with the drug response variability of bortezomib, leading to various disease prognoses [10]. The relationships between the heterogeneity of drug response in bortezomib or its combined therapy with the genomic background of multiple myeloma patients have been investigated [2, 10]. Mulligan et al. [10] generated gene expression data from a national and international phase 2 and 3 clinical trials of bortezomib to develop a genomic classifier for prediction of drug response in relapsed MM. Terragna et al. [2] analyzed the gene expression from MM patients to explore predictors of bortezomib-thalidomide-dexamethasone (VTD) first-line therapy. According to European Group for Bone Marrow Transplantation criteria, drug responses in MM were classified as achieving complete response (CR), partial response (PR), minimal response (MR), no change (NC) and Progressive Disease (PD) [21]. However, in Mulligan et al. [10], the five-level ordinal drug response was categorized as a binary response where only responsive and non-responsive groups were considered. Terragna et al. [2] focused on CR vs non-CR groups by converting the ordinal outcome to a binary outcome. To provide more informative prediction and more efficiently identify biomarkers, it is desirable to analyze multi-level drug response, rather than combining the response to two groups. To address this shortcoming in the previous analyses, we here present a novel approach to identify significantly associated biomarkers and develop genomic classifier using hierarchical ordinal logistic regression. We apply our approach to two public available datasets [2, 10]. Our results show that our hierarchical ordinal regression approach is able to identify genes associated with the multi-level response and to generate predictive models for predicting the multi-level response.

## Methods

### Datasets acquisition for ordinal response prediction

The gene expression datasets analyzed to predict the drug response were acquired from two independent clinical trials. The two datasets are publically available from GEO under accession number [GEO: GSE9782] and [GEO: GSE68871]. They were originally published in Mulligan et al. [10] and Terragna et al. [2]. Mulligan et al. [10] recruited patients (*n = 169*) with relapsed myeloma enrolled in phase 2 and phase 3 clinical trials of bortezomib, whose pretreated tumor samples were further analyzed for genomic profiling with consent. Myeloma samples were collected prior to enrollment in clinical trials of bortezomib and samples were subject to replicate gene expression profiling using the Affymetrix 133A microarray (22,283 probes). Terragna et al. [2] focused primarily on treating the new MM patients (*n = 118*) with the induction therapy of VTD. The gene expression profiling (54,677 probes) was carried out in the Affymetrix Human Genome U133 Plus 2.0 Array. We standardized all the probes for statistical analysis in both datasets.

### Outcome definitions

The original datasets contained drug response and overall survival as two outcomes. We here focused on developing genomic classifier for the drug response. In Mulligan et al. [10], patients were classified as achieving complete response (CR), partial response (PR), minimal response (MR), no change (NC) and Progressive Disease (PD) according to European Group for Bone Marrow Transplantation criteria [21]. In Terragna et al. [2], patients’ drug responses were classified as five categories: complete response (CR), near complete response (nCR), very good partial response (VGPR), partial response (PR) and stable disease (SD). For both datasets, the five-level ordinal drug response was used in our analysis. In the meantime, we combined the five-level drug response to a new three-level drug response in both datasets to avoid low frequencies in certain levels. For Mulligan et al. [10], we combined PD and NC to a new level, and PR and MR as another new level. For Terragna et al. [2], we combined SD and PR as a new level, and VGPR and nCR as another new level. Both the original five-level and the new three-level outcomes were separately analyzed for these two datasets.

### Ordinal drug response prediction modeling

Let *y*_*i*_ be the ordinal outcome for which there exists a clear ordering of the response categories, and *X*_*ij*_ the gene expression profile for the *i*th individual and *j*th probe in the data of sample size *n* with a total number *J* of probes. For notational convenience, we code the ordinal outcome as the integers 1, 2, ···, *K*, with *K* being the number of categories.

#### Univariate ordinal logistic regression to rank top probes

It will not be efficient to include all the genes in a predictive model, due to the large number of genes. We thus first use the univariate ordinal logistic regression to filter top *q* associated probes of the gene expression profile with the ordinal outcome. For the *j*-th gene expression *X*_*ij*_, the univariate ordinal logistic regression is expressed as:

$$ \Pr \left({y}_i=k\right)=\left\{\begin{array}{l}1-{\mathrm{logit}}^{-1}\left({X}_{ij}\alpha -{c}_{1j}\right)\kern9em \mathrm{if}\kern0.5em k=1\\ {}{\mathrm{logit}}^{-1}\left({X}_{ij}\alpha -{c}_{\left(k-1\right)j}\right)-{\mathrm{logit}}^{-1}\left({X}_{ij}\alpha -{c}_{kj}\right)\kern1.25em \mathrm{if}\kern0.5em 1<k<K\\ {}{\mathrm{logit}}^{-1}\left({X}_{ij}\alpha -{c}_{\left(k-1\right)j}\right)\kern9.25em \mathrm{if}\kern0.5em k=K\ \end{array}\right. $$

where *c*_*kj*_ denoted cut-points or thresholds, are constrained to increase, *c*_1*j*_ < ⋯ < *c*_(*K* − 1)*j*_. We then select the top *q* associated probes based on the *p*-value for testing the hypothesis H_0_: *α* = 0.

#### Predictive modeling for genomic classifiers

We use all the *q* selected probes to build a multivariable ordinal model for predicting the multi-level response, i.e.

$$ \Pr \left({y}_i=k\right)=\left\{\begin{array}{l}1-{\mathrm{logit}}^{-1}\left({X}_i\beta -{c}_1\right)\kern8.5em \mathrm{if}\kern0.5em k=1\\ {}{\mathrm{logit}}^{-1}\left({X}_i\beta -{c}_{k-1}\right)-{\mathrm{logit}}^{-1}\left({X}_{ij}\beta -{c}_k\right)\kern1.25em \mathrm{if}\kern0.5em 1<k<K\\ {}{\mathrm{logit}}^{-1}\left({X}_i\beta -{c}_{k-1}\right)\kern9em \mathrm{if}\kern0.5em k=K\ \end{array}\right. $$

where the vector *X*_*i*_ includes the expression measures of the *q* genes, and *β* = (*β*_1_, · ··, *β*_*q*_)^T^ is a vector of the effects. With hundreds or tens of correlated top associated probes, however, the standard ordinal logistic regression may fail, due to the non-identifiability and overfitting. To overcome the problems, we use an appropriate prior distribution to constrain the coefficients to lie in reasonable ranges and allow the model to be reliably fitted and to identify important predictors [22, 23]. We employ the commonly used Cauchy prior distribution on the coefficients in the ordered logistic regression:

$$ p\left({\beta}_j\right)=\mathrm{Caucy}\left(0,s\right)=\frac{1}{\pi s}\frac{1}{1+{\beta}_j^2/{s}^2} $$

The scale parameter s controls the amount of shrinkage in the coefficient estimate; smaller s induces stronger shrinkage and forces more coefficients towards zero. For the cut-points parameters, we use a uniform prior. We have developed a quasi-Newton algorithm (BFGS) for fitting the hierarchical ordinal model by finding the posterior mode of the parameters (β, c), i.e., estimating the parameters by maximizing the posterior density.

Our algorithm has been implemented in our R package BhGLM, which is freely available from the website http://www.ssg.uab.edu/bhglm/ and the public GitHub repository https://github.com/abbyyan3/BhGLM that includes R codes for examples.

#### Assessing the performance of a fitted hierarchical ordinal logistic regression

After fitting a hierarchical ordinal model, we obtain the estimate ($\widehat{\beta},\widehat{c}$) and can estimate the probabilities: ${p}_{ik}=\Pr \left({y}_i=k|{X}_i\widehat{\beta},\widehat{c}\right),i=1,\cdots, n;k=1,\cdots, K$. Denote *y*_*ik*_ = I(*y*_*i*_ = *k*) as the binary indictor response for the *k*-th category. We can evaluate the performance using several measures:

1. *Deviance*: $d=-2{\sum}_{i=1}^n\log {p}_{ik}$. Deviance measures the overall quality of a fitted model;
2. *AUC* (area under the ROC curve). We can calculate AUC for the *k*-th category using {*y*_*ik*_, *p*_*ik*_: *i* = 1, ···, *n*} as usual. Then the AUC for all the categories is defined as$\frac{1}{K}{\sum}_{k=1}^K{AUC}_k$.
3. *MSE* (mean squared error). MSE is defined as: $MSE=\frac{1}{K}{\sum}_{k=1}^K\left[\frac{1}{n}{\sum}_{i=1}^n{\left({y}_{ik}-{p}_{ik}\right)}^2\right]$.
4. *Misclassification*. The misclassification is defined as: $MIS=\frac{1}{K}{\sum}_{k=1}^K\left[\frac{1}{n}{\sum}_{i=1}^nI\left(|{y}_{ik}-{p}_{ik}|>0.5\right)\right]$, where *I*(| *y*_*ik*_ − *p*_*ik*_| >0.5) = 1 if ∣*y*_*ik*_ − *p*_*ik*_ ∣  > 0.5, and *I*(| *y*_*ik*_ − *p*_*ik*_| >0.5) = 0 if ∣*y*_*ik*_ − *p*_*ik*_ ∣  ≤ 0.5;

To evaluate the predictive performance of the model, we use the pre-validation method, a variant of cross-validation [24, 25], by randomly splitting the data to *H* subsets of roughly the same size, and using (*H* – 1) subsets to fit a model. We then calculate the measures described above with *h*th subset and cycle through all *H* subsets to get the averaged measurements to evaluate the predictive performance. To get stable results, we can run *H*-fold cross-validation multiple times and use the average of the measure over the repeats to assess the predictive performance. We also can use leave-one-out cross-validation (i.e., *H = n*) to obtain unique result. In this study, 10-fold cross-validation with 10 repeats and leave-one-out cross-validation were both utilized. Deviance AUC, MSE and misclassification rate were all reported.

#### Selecting optimal scale values

The performance of the hierarchical ordinal model can depend on the scale parameter in the Cauchy prior. We fit a sequence of models with different scales ranging from 0.01 to 1 by 0.01, from which we can choose an optimal one based on the criteria described above.

#### Selecting the optimal number of *q* probes

To select an optimal number of top *q* probes, we fit a sequence of models with a different number of probes with the options from 30 and 50 to 500 by 50. The number *q* will be determined based on the predictive performance of the hierarchical ordinal logistic regression by evaluating the deviance of the models. The chosen top *q* probes will be identified as associated significant biomarkers and present in heatmaps for visual examination.

## Results

### Data summary

There were 169 samples analyzed in the dataset, with a total of 22,283 gene expression probes in Mulligan et al. [10]. In Terragna et al. [2], we analyzed 118 samples with a total of 54,677 gene expression probes. The details of both studies and the frequencies of the five-level ordinal drug response outcomes were summarized in Table 1. To avoid low frequencies in some levels for both datasets, we combined the five-level drug response to a new three-level drug response. By combining drug response in Mulligan et al. [10] as the new three-level ordinal outcome, there were 73 patients having a response as PD or NC, 55 patients having a response as MR or PR and 41 patients having a response as CR. By combining drug response in Terragna et al. [2] as the new three-level ordinal outcome, there were 49 patients having a response as SD or PR, 54 patients having a response as VGPR or nCR and 15 patients having a response as CR.

**Table 1:** Summary of studies and frequency table for original ordinal outcome in both studies

Study	Mulligan et al. [10]	Terragna et al. [2]
Treatment	Bortezomib	VTD
Number of Samples	169	118
Number of Probes	22,283	54,677
Patients Population	Relapsed MM	New-Diagnosis
	Progressive Disease (PD)	Stable Disease (SD)
	13 (7.70%)	7 (5.93%)
	No Change (NC)	Partial Response (PR)
	60 (35.50%)	42 (35.59%)
	Minimal Response (MR)	Very good partial response (VGPR)
	12 (7.10%)	40 (33.90%)
	Partial Response (PR)	near Complete Response (nCR)
	43 (25.44%)	14 (11.87%)
	Complete Response (CR)	Complete Response (CR)
	41 (24.26%)	15 (12.71%)

### Predictive genomic classifiers analysis

For the original ordinal drug response outcome in Mulligan et al. [10], we first filtered probes based on all the options from top 30 and then 50 probes to top 500 probes by 50 probes. Based on the predictive performance of all the 11 models (Table 2) evaluated with 10-fold cross-validation with 10 repeats, it showed that the best predictive model would include all 450 top probes for smallest deviance. However, for the final predictive model, we included top 50 probes as predictors with a balance between a reasonable decrease in deviance and simplicity in predictive model for clinical application. The prior scale in the final model was chosen at 0.14. Deviance of the final model was 441.919 and AUC was 0.632; while MSE was 0.136 and misclassification rate was 0.189. For the new combined ordinal drug response outcome in Mulligan et al. [10], we filtered probes based on all the options from top 30 and then 50 probes to top 500 probes by 50 probes. Based on the predictive performance of all the 11 models (Table S1 [See Additional file 1]) evaluated by 10-fold cross-validation with 10 repeats, it showed that the best predictive model included all 400 top probes for smallest deviance. However, for the final predictive model, we included top 50 probes as predictors with a balance between a reasonable decrease in deviance and simplicity in predictive model for clinical application. The prior scale in the final model was chosen at 0.16. For the final model, deviance was 316.118, AUC was 0.696, MSE was 0.190 and misclassification rate was 0.273. Comparing the predictive modeling performance between five-level ordinal drug response and the new combined ordinal drug response, AUC increased by 0.060 and deviance decreased by 125.801 for the combined ordinal outcome with a trade-off in MSE increasing by 0.054 and misclassification rate increasing by 0.084.

**Table 2:** Summary of predictive performance using different number of top probes for drug response prediction (five levels) in two studies

		10 fold with 10 repeats cross-validation				Leave one out cross-validation			
Number of top genes	Prior Scale	Deviance	AUC	MSE	misclassification	Deviance	AUC	MSE	misclassification
Mulligan et al. [10] (Five Level Drug response Outcome)									
30	0.4	452.868	0.616	0.140	0.204	452.805	0.605	0.140	0.201
50	0.14	441.919	0.632	0.136	0.189	442.235	0.618	0.137	0.194
100	0.14	439.464	0.634	0.136	0.190	438.839	0.625	0.136	0.188
150	0.15	431.669	0.657	0.133	0.185	434.309	0.645	0.134	0.186
200	0.15	425.471	0.673	0.132	0.186	427.950	0.662	0.133	0.189
250	0.15	428.840	0.669	0.133	0.187	431.478	0.661	0.133	0.189
300	0.15	437.913	0.660	0.135	0.189	443.893	0.642	0.137	0.194
350	0.15	425.215	0.690	0.133	0.185	430.448	0.679	0.135	0.186
400	0.09	411.556	0.692	0.130	0.181	407.755	0.690	0.129	0.178
450	0.09	410.313	0.700	0.129	0.179	402.852	0.704	0.127	0.179
500	0.14	426.196	0.705	0.134	0.194	434.344	0.691	0.138	0.209
Terragna et al. [2] (Five Level drug response Outcome)									
30	0.95	270.440	0.776	0.126	0.188	266.318	0.780	0.126	0.186
50	0.17	270.285	0.755	0.128	0.195	267.102	0.764	0.127	0.192
100	0.17	267.890	0.757	0.126	0.185	264.300	0.764	0.124	0.183
150	0.26	277.956	0.766	0.129	0.190	276.060	0.770	0.130	0.197
200	0.26	277.353	0.767	0.130	0.187	275.197	0.765	0.130	0.188
250	0.26	274.008	0.775	0.128	0.187	265.904	0.778	0.127	0.169
300	0.23	273.854	0.779	0.128	0.185	268.504	0.779	0.126	0.180
350	0.23	272.087	0.776	0.126	0.174	271.819	0.779	0.124	0.169
400	0.12	264.856	0.769	0.126	0.182	258.087	0.774	0.121	0.173
450	0.16	263.406	0.779	0.124	0.176	258.174	0.785	0.121	0.173
500	0.16	259.613	0.789	0.122	0.166	252.812	0.796	0.118	0.159

For the original ordinal drug response outcome in Terragna et al. [2], we also filtered probes based on all the options from top 30 and then 50 probes to top 500 probes by 50 probes. Based on the predictive performance of all the 11 models (Table 2), it showed that the best predictive model included all 500 top probes for smallest deviance. However, for the final predictive model, we included top 30 probes as predictors with a balance between a comparable low deviance and simplicity in predictive model for clinical application. The prior scale in the final model was chosen at 0.95. For the final model, deviance was 270.440, AUC was 0.776, MSE was 0.126 and misclassification rate was 0.188. For the new combined ordinal drug response outcome in Terragna et al. [2], we filtered probes based on all the options from top 30 and then 50 probes to top 500 probes by 50 probes. Based on the predictive performance of all the 11 models (Additional file 1: Table S1), it showed that the best predictive model included all 500 top probes for smallest deviance. However, for the final predictive model, we included top 50 probes as predictors with a balance between a comparable low deviance and simplicity in predictive model for clinical application. The prior scale in the final model was chosen at 0.26. Deviance of the final model was 167.130 and AUC was 0.800; while MSE was 0.152 and misclassification rate was 0.233. We compared the predictive performance of all models using 10-fold cross-validation with 10 repeats and leave one out cross-validation, which lead to similar results. The results were shown in Table 2 and Additional file 1: Table S1. Comparing the predictive modeling performance between five-level ordinal drug response and the new combined ordinal drug response, AUC increased by 0.024 and deviance decreased by 103.310 for the combined ordinal outcome with a trade-off in MSE increasing by 0.026 and misclassification rate increasing by 0.045.

### Genes identification

**Fig. 1:**
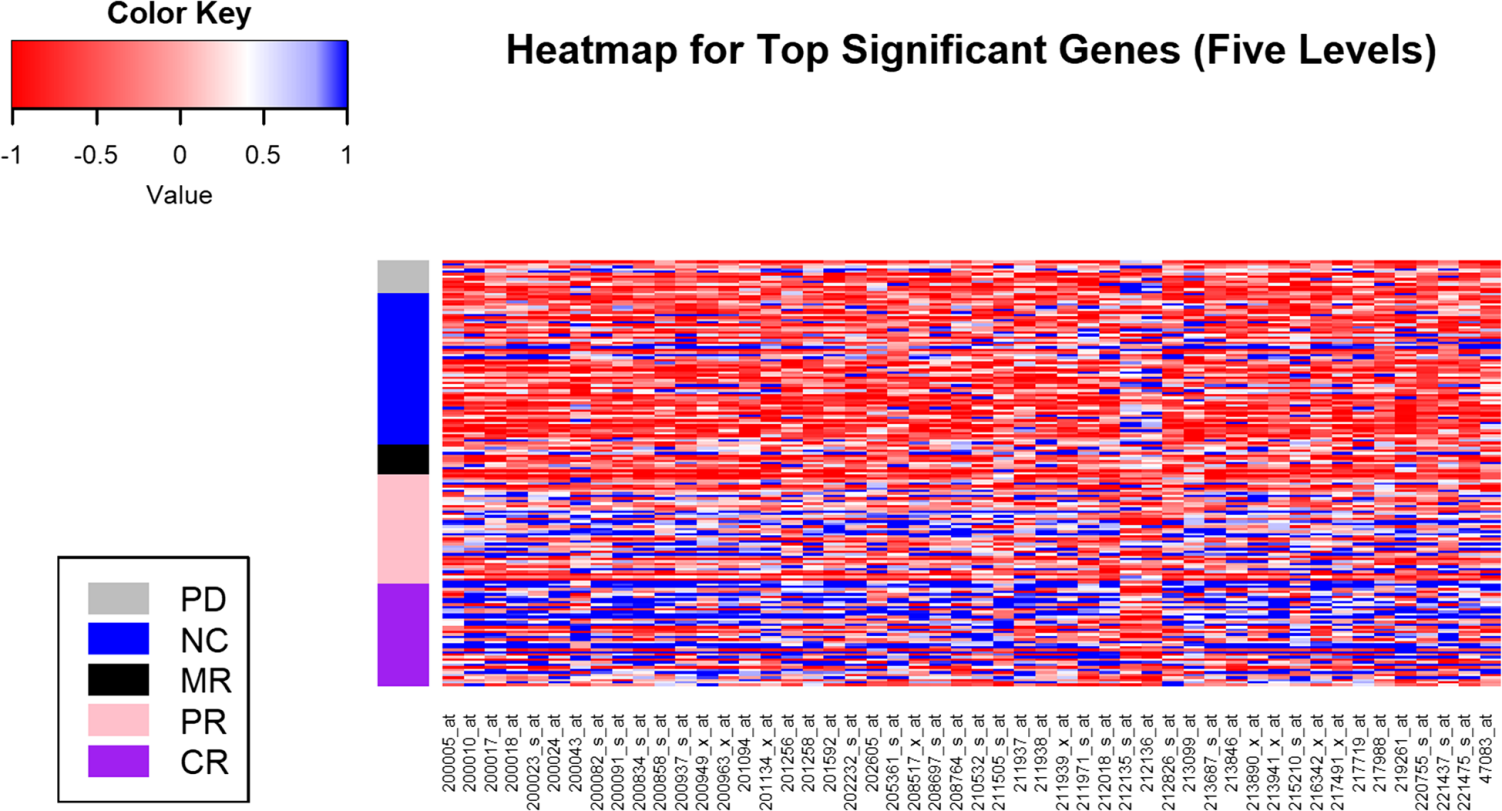
Heatmap with Top 50 Significantly Probes with Drug response (Five Levels) in Mulligan et al. [10]. A heatmap for the gene expression of selected top significant 50 probes which were used as predictive genomic factors for the five-level ordinal drug response from Mulligan et al. [10]. The bottom of the heatmap presents the names of the 50 probes; while the left side color bar stands for five-level ordinal drug response, including complete response (CR), partial response (PR), minimal response (MR), no change (NC) and progressive disease (PD)

To visualize the selected significant probes and its relationship with the clinical outcome in Mulligan et al. [10], a heatmap was presented in Fig. 1 with the top 50 significant probes which were used as predictive genomic factors for the five-level ordinal drug response. The top 50 significant probes represent genes of known function. Most of the probes are overexpressed in patients with PR or CR, which covers various functions including ribosomal protein (RPL11, RPL15, RPS7, RPS13), mitochondrial (COX7C), eukaryotic translation initiation factors (EIF3D, EIF3E, EIF3F, EIF3H) genes. Two of the probes are under-expressed in patients achieving PR or CR, which represent the gene function as ATPase plasma membrane Ca2+ transporting 4 (ATP2B4). For the three-level ordinal drug response, a heatmap was presented in Figure S1 [See Additional file 2] with the top 50 significant probes which were used as predictive genomic factors in our final predictive model. The top 50 probes for three-level drug response overlapped with most of the top 50 probes for five-level drug response. Only a few probes represent different genes of functions, including eukaryotic translation elongation factor (EEF2), chloride voltage-gated channel (CLCN3), abhydrolase domain containing (ABHD14A).

**Fig. 2:**
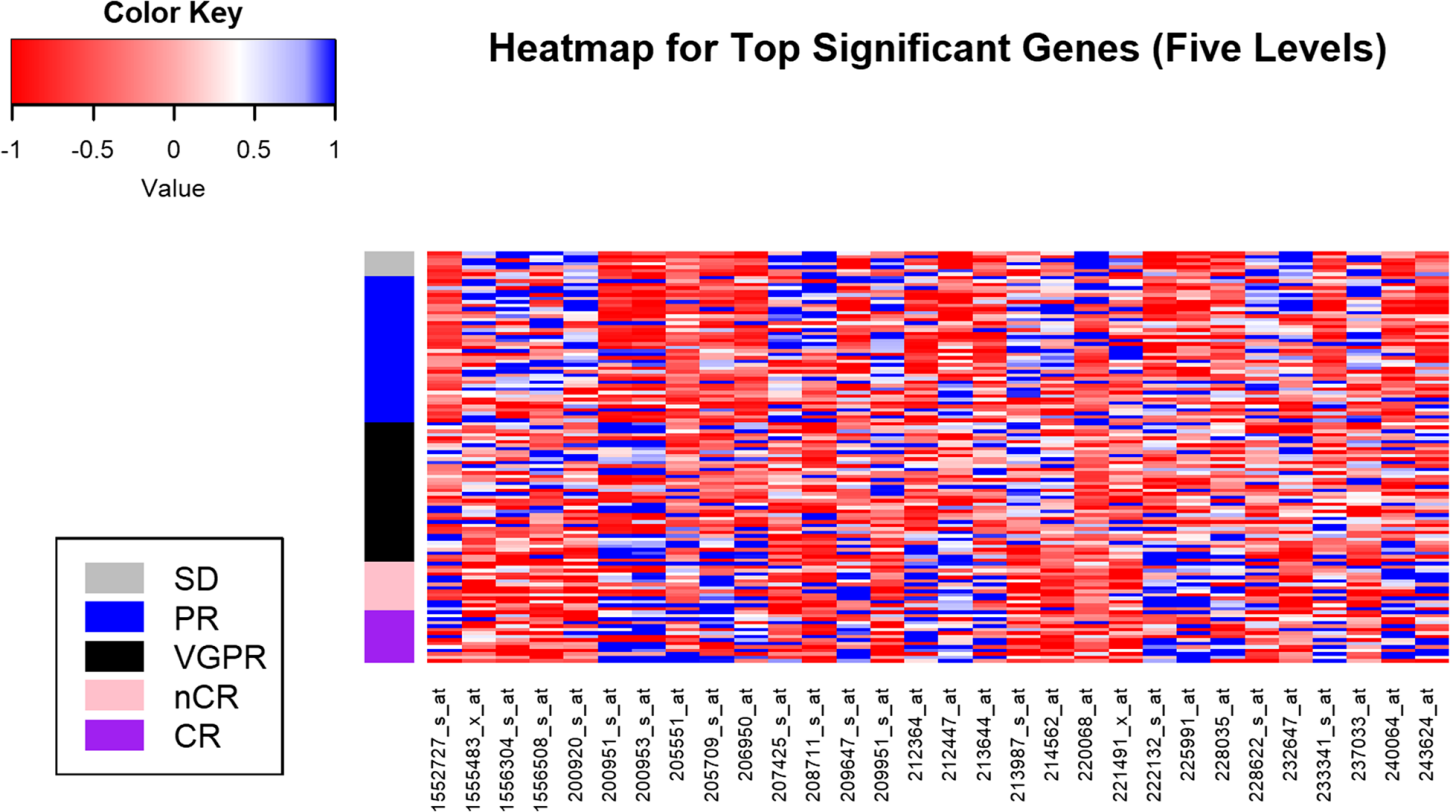
Heatmap with Top 30 Significantly Probes with Drug response (Five Levels) in Terragna et al. [2]. A heatmap for the gene expression of selected top significant 30 probes which were used as predictive genomic factors for the five-level ordinal drug response from Terragna et al. [2]. The bottom of the heatmap presents the names of the 30 probes; while the left side color bar stands for five-level ordinal drug response, including complete response (CR), near complete response (nCR), very good partial response (VGPR), partial response (PR) and stable disease (SD)

To visualize the selected significant probes and its relationship with the clinical outcome in Terragna et al. [2], a heatmap was presented in Fig. 2 with the top 30 significant probes which were used as predictive genomic factors for the five-level ordinal drug response. The top 30 probes in this dataset differentiated with similar chance to over-express or down-express, which also cover various functions including BTG anti-proliferation factor 1 (BTG1), CDP-diacylglycerol synthase 1 (CDS1), RNA polymerase I subunit B (POLR1B), acylglycerol kinase (AGK), cyclin D1 (CCND1), cyclin D2 (CCND2), major histocompatibility complex, class II, DQ beta 1 (HLA-DQB1), mitogen-activated protein kinase 7 (MAP2K7) and suppressor of cytokine signaling 5 (SOCS5) genes. For the three-level ordinal drug response, a heatmap was presented in Figure S2 [See Additional file 3] with the top 50 significant probes which were used as predictive genomic factors in our final predictive model. The top 30 probes for three-level drug response overlapped with most of the top 50 probes for five-level drug response. Only a few probes represent different genes of functions, including SRC proto-oncogene, non-receptor tyrosine kinase (SRC), TNF receptor superfamily member 13C (TNFRSF13C) and checkpoint kinase 1 (CHEK1).

## Discussion

It is highly important to identify genetic biomarkers to predict drug response with a narrow therapeutic index [19, 26]. Chemotherapeutic agents are medications in that category, since the response is variable with potentially lethal side effects [19, 26]. Many studies have been conducted and a large number of biomarkers have been reported [19]. However, a complex outcome, such as drug response, is generally affected by many genomic and environmental factors [19]. Thus, it is desirable that a predictive procedure will possess the capability to consider mutual effects of various biomarkers for drug response [19].

Another potential issue is that the multi-level ordinal drug response is usually recoded in clinical practice. For analytic simplicity, however, such multi-level ordinal outcome is usually combined as just two levels, as in the original papers [2, 10] that we reanalyzed in this study. However, this strategy not only risks both the loss of information in the data and arbitrary to select the recode strategy, but also cannot provide informative prediction [27].

We here utilized a more efficient approach by combining the standard ordinal logistic regression and the hierarchical modeling. Our method can jointly analyze numerous variables for detecting important predictors and for predicting multi-level drug response. We applied our method to reanalyze two publicly available clinical trial datasets, which assessed response to bortezomib in relapsed MM patients [10] and VTD in newly diagnosed MM patients [2]. The original studies both treated the five-level ordinal drug responses as binary responses. To address the drawback of the potential loss of information from recoding, we reanalyzed the datasets by using the original ordinal drug responses. To avoid low frequencies in several levels of the five-level drug responses, we redefined the five-level drug response as a three-level ordinal drug response in both datasets. The results reveal that the predictive performance from VTD in new MM patients is more powerful than treating relapsed MM patients with bortezomib alone. Meanwhile, comparing the analysis results between five-level ordinal drug response and reduced three-level ordinal drug response, AUC increased and deviance decreased for the combined ordinal outcome with a trade-off in MSE and misclassification rate. Our analyses show that the combining ordinal outcome could result in higher MSE and misclassification rate, thus, potential loss of information and misleading interpretation. Although we only compared the five-level ordinal outcome with three-level ordinal outcome, it is anticipated that similar differences will exist if compared with binary outcome. It also implies that the original approach to analyze ordinal outcome as binary outcome will possibly lead to information loss.

Furthermore, we identified probes that represent genes of known function. In Mulligan et al. [10], for both five level and three level ordinal drug response, most of the top significant probes are overexpressed in patients with PR or CR, including ribosomal protein (RPL11, RPL15, RPS7, RPS13), mitochondrial (COX7C), eukaryotic translation initiation factors (EIF3D, EIF3E, EIF3F, EIF3H) genes. Among them, ribosomal protein has been investigated by multiple studies to show that mutations in ribosomal protein genes have been found in endometrial cancer (RPL22), T-cell acute lymphoblastic leukemia (RPL10, RPL5 and RPL11), chronic lymphocytic leukemia (RPS15), colorectal cancer (RPS20), and glioma (RPL5) [28]. Moreover, it has also been discussed that eukaryotic initiation factors (EIFs) play an important role in translation initiation and protein synthesis which could alter angiogenesis, tumor development, and apoptosis in cancer progression [29]. Two of the probes are under-expressed in patients achieving PR or CR, which represent the gene ATP2B4. ATP2B4 plays a critical role in intracellular calcium homeostasis by regulating the enzymes to remove bivalent calcium ions from eukaryotic cells against very large concentration gradients [30]. In Terragna et al. [2], we carried a function enrichment analysis to identify the functional annotation of the top probes with KEGG [31] using the Bioinformatics tool *DAVID* [32, 33]. The top 30 probes for the five-level ordinal drug response also cover various gene functions which belong to multiple important pathways, e.g., Metabolic pathways, p53 signaling pathway, PI3K-Akt signaling pathway, AMPK signaling pathway, Wnt signaling pathway, Jak-STAT signaling pathway, Viral carcinogenesis and MAPK signaling pathway. For the three-level ordinal drug response, the top 50 significant probes cover similar functions as the top 30 probes for the five-level ordinal drug response, with several additional functions such as Cytokine-cytokine receptor interaction, NF-kappa B signaling pathway, Intestinal immune network for IgA production, HTLV-I infection and Primary immunodeficiency. This suggests that the probes we identified are correlated biologically. Based on the functional enrichment analysis results, the probes could be grouped to multiple pathways. One plausible solution is to utilize a pathway-structured model to incorporate that biological information into the predictive model to include more probe information into the prediction, which will be considered in our further work.

Although the predictive classifier and genetic biomarkers described here are promising, further research is necessary to assess the relevance of these genomic predictors with more data from other trials or other trials with novel or multi-agent therapy. Our analysis strategy is directly applicable to new data with bortezomib or other therapies in multiple myeloma for patients with newly diagnosed or relapsed cancer. This analysis will help to quickly identify the patient groups that could benefit from the proposed drug therapy or in need of other novel therapies.

## Conclusions

We propose a novel method to directly analyze the multi-level drug response, rather than combining the response to two groups. Our method employs a hierarchical ordinal logistic model with the heavy-tailed Cauchy prior on the coefficients. The proposed method allows us to jointly fit numerous correlated predictors and thus build efficient models for predicting the multi-level drug response. The predictive model for the multi-level drug response can be more informative than the previous approaches. Thus, the proposed approach provides a powerful tool for predicting multi-level drug response and has important impact on cancer studies.

## Additional files

Additional file 1: **Table S1.** Summary of predictive performance using different number of top probes for drug response prediction (three levels) in two studies. (DOCX 21 kb)

Additional file 2: **Figure S1.** Heatmap with Top 50 Significantly Probes with Drug response (Three Levels) in Mulligan et al. [10]. (DOCX 77 kb)

Additional file 3: **Figure S2.** Heatmap with Top 50 Significantly Probes with Drug response (Three Levels) in Terragna et al. [2]. (DOCX 72 kb)

## Abbreviations

AUC: Area under the ROC curve
BFGS: Quasi-Newton algorithm
CR: Complete response
EIFs: Eukaryotic initiation factors
IgH: Immunoglobulin heavy
MM: Multiple Myeloma
MR: Minimal response
MSE: Mean squared error
NC: No change
nCR: Near complete response
PD: Progressive disease
PR: Partial response
SD: Stable disease
VGPR: Very good partial response
VTD: Bortezomib-thalidomide-dexamethasone (VTD)
