# Supplementary material for: Predicting multi-level drug response with gene expression profile in multiple myeloma using hierarchical ordinal regression
Source: BMC Cancer. 2018 May 10;18:551. doi: 10.1186/s12885-018-4483-6 (PMC5946496; doi:10.1186/s12885-018-4483-6)
Supplement: Supplementary file 1 — Table S1. Summary of predictive performance using different number of top probes for drug response prediction (three levels) in two studies. (DOCX 21 kb) [file 12885_2018_4483_MOESM1_ESM.docx]

**Table S1.** Summary of predictive performance using different number of top probes for drug response prediction (three levels) in two studies.

|  |  | 10 Fold with 10 Repeats Cross Validation | | | | Leave One Out Cross Validation | | | |
| --- | --- | --- | --- | --- | --- | --- | --- | --- | --- |
| Number of top genes | Prior Scale | Deviance | AUC | MSE | misclassification | Deviance | AUC | MSE | Misclassification |
| Mulligan et al. (10) (Three Level Outcome) | | | | | | | | | |
| 30 | 0.4 | 319.459 | 0.692 | 0.192 | 0.286 | 319.730 | 0.690 | 0.192 | 0.286 |
| 50 | 0.16 | 316.118 | 0.696 | 0.190 | 0.273 | 316.303 | 0.693 | 0.190 | 0.272 |
| 100 | 0.21 | 309.989 | 0.716 | 0.185 | 0.278 | 310.457 | 0.711 | 0.186 | 0.260 |
| 150 | 0.19 | 305.666 | 0.726 | 0.183 | 0.286 | 308.387 | 0.718 | 0.185 | 0.290 |
| 200 | 0.15 | 290.714 | 0.750 | 0.176 | 0.277 | 295.732 | 0.741 | 0.179 | 0.282 |
| 250 | 0.15 | 293.980 | 0.746 | 0.178 | 0.273 | 301.352 | 0.731 | 0.183 | 0.270 |
| 300 | 0.15 | 296.882 | 0.745 | 0.178 | 0.273 | 292.503 | 0.751 | 0.175 | 0.254 |
| 350 | 0.15 | 299.856 | 0.742 | 0.180 | 0.273 | 298.527 | 0.749 | 0.179 | 0.264 |
| 400 | 0.09 | 286.815 | 0.757 | 0.173 | 0.256 | 284.755 | 0.755 | 0.173 | 0.258 |
| 450 | 0.09 | 287.807 | 0.754 | 0.174 | 0.257 | 286.292 | 0.751 | 0.175 | 0.260 |
| 500 | 0.09 | 288.178 | 0.754 | 0.174 | 0.258 | 283.473 | 0.757 | 0.173 | 0.258 |
| Terragna et al. (2) (Three Level Outcome) | | | | | | | | | |
| 30 | 0.26 | 169.341 | 0.802 | 0.150 | 0.221 | 166.872 | 0.811 | 0.147 | 0.212 |
| 50 | 0.26 | 167.130 | 0.800 | 0.152 | 0.233 | 163.637 | 0.808 | 0.149 | 0.226 |
| 100 | 0.35 | 173.866 | 0.801 | 0.154 | 0.226 | 172.772 | 0.808 | 0.152 | 0.226 |
| 150 | 0.35 | 176.737 | 0.798 | 0.155 | 0.225 | 176.668 | 0.802 | 0.154 | 0.229 |
| 200 | 0.26 | 170.301 | 0.798 | 0.158 | 0.245 | 172.170 | 0.798 | 0.162 | 0.266 |
| 250 | 0.27 | 165.507 | 0.814 | 0.150 | 0.232 | 167.312 | 0.812 | 0.152 | 0.260 |
| 300 | 0.3 | 155.106 | 0.838 | 0.140 | 0.220 | 155.246 | 0.840 | 0.140 | 0.251 |
| 350 | 0.34 | 152.087 | 0.847 | 0.136 | 0.199 | 153.380 | 0.848 | 0.137 | 0.189 |
| 400 | 0.34 | 146.268 | 0.851 | 0.130 | 0.178 | 146.917 | 0.853 | 0.130 | 0.175 |
| 450 | 0.25 | 145.343 | 0.849 | 0.130 | 0.184 | 145.761 | 0.850 | 0.131 | 0.192 |
| 500 | 0.25 | 144.767 | 0.854 | 0.129 | 0.176 | 145.762 | 0.853 | 0.129 | 0.189 |
