# Supplementary material for: Predicting multi-level drug response with gene expression profile in multiple myeloma using hierarchical ordinal regression
Source: BMC Cancer. 2018 May 10;18:551. doi: 10.1186/s12885-018-4483-6 (PMC5946496; doi:10.1186/s12885-018-4483-6)
Supplement: Supplementary file 2 — Figure S1. Heatmap with Top 50 Significantly Probes with Drug response (Three Levels) in Mulligan et al. [10]. (DOCX 77 kb) [file 12885_2018_4483_MOESM2_ESM.docx]

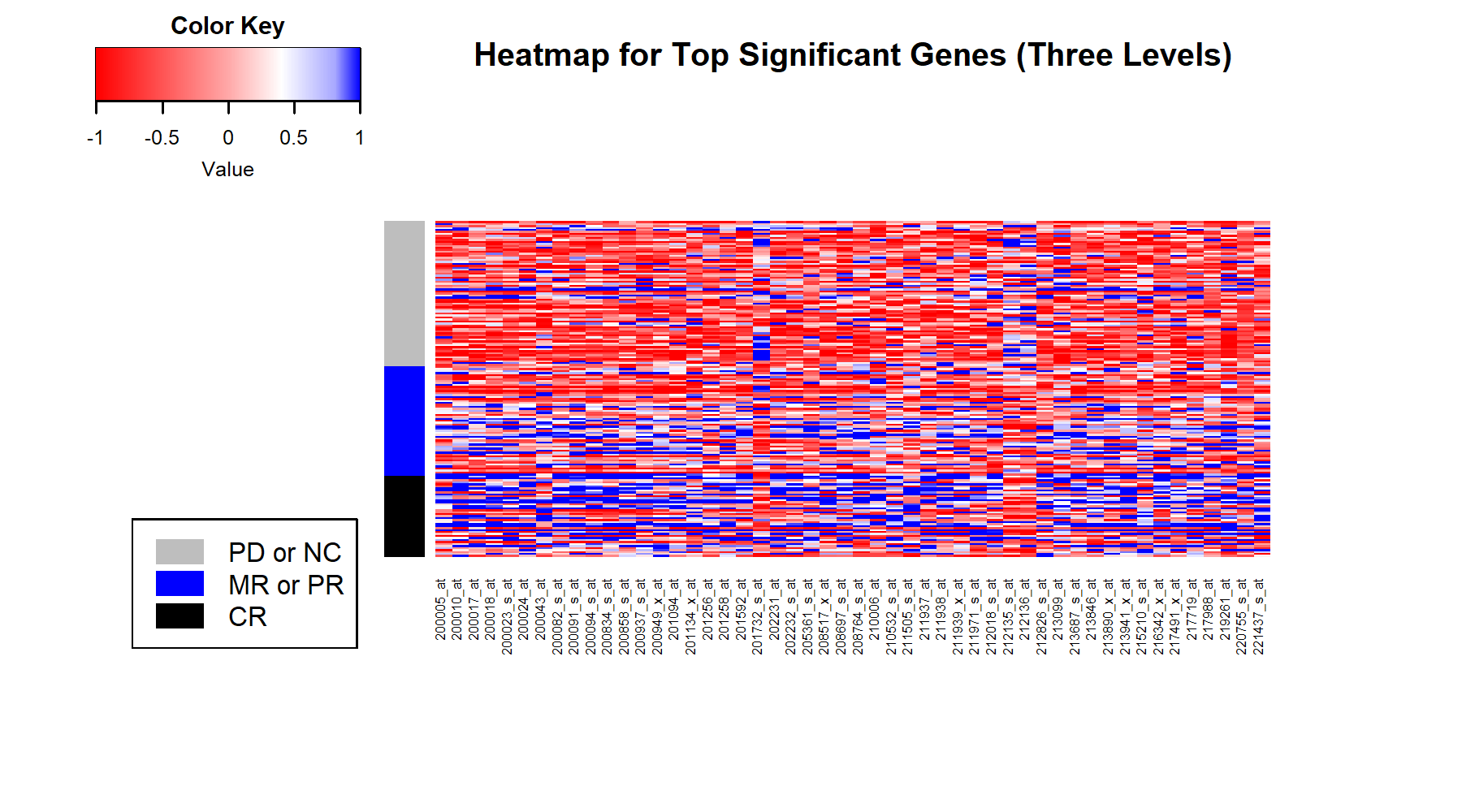


**Figure S1.** Heatmap with Top 50 Significantly Probes with Drug response (Three Levels) in Mulligan et al. [(10)](#_ENREF_10).

Figure S.1 is a heatmap for the gene expression of selected top significant 50 probes which were used as predictive genomic factors for the three-level ordinal drug response from Mulligan et al. [(10)](#_ENREF_10). The bottom of the heatmap presents the names of the 50 probes; while the left side color bar stands for three-level ordinal drug response, including complete response (CR), minimal response or partial response (MR or PR), progressive disease or no change (PD or NC).
