# Supplementary material for: Predicting multi-level drug response with gene expression profile in multiple myeloma using hierarchical ordinal regression
Source: BMC Cancer. 2018 May 10;18:551. doi: 10.1186/s12885-018-4483-6 (PMC5946496; doi:10.1186/s12885-018-4483-6)
Supplement: Supplementary file 3 — Figure S2. Heatmap with Top 50 Significantly Probes with Drug response (Three Levels) in Terragna et al. [2]. (DOCX 72 kb) [file 12885_2018_4483_MOESM3_ESM.docx]

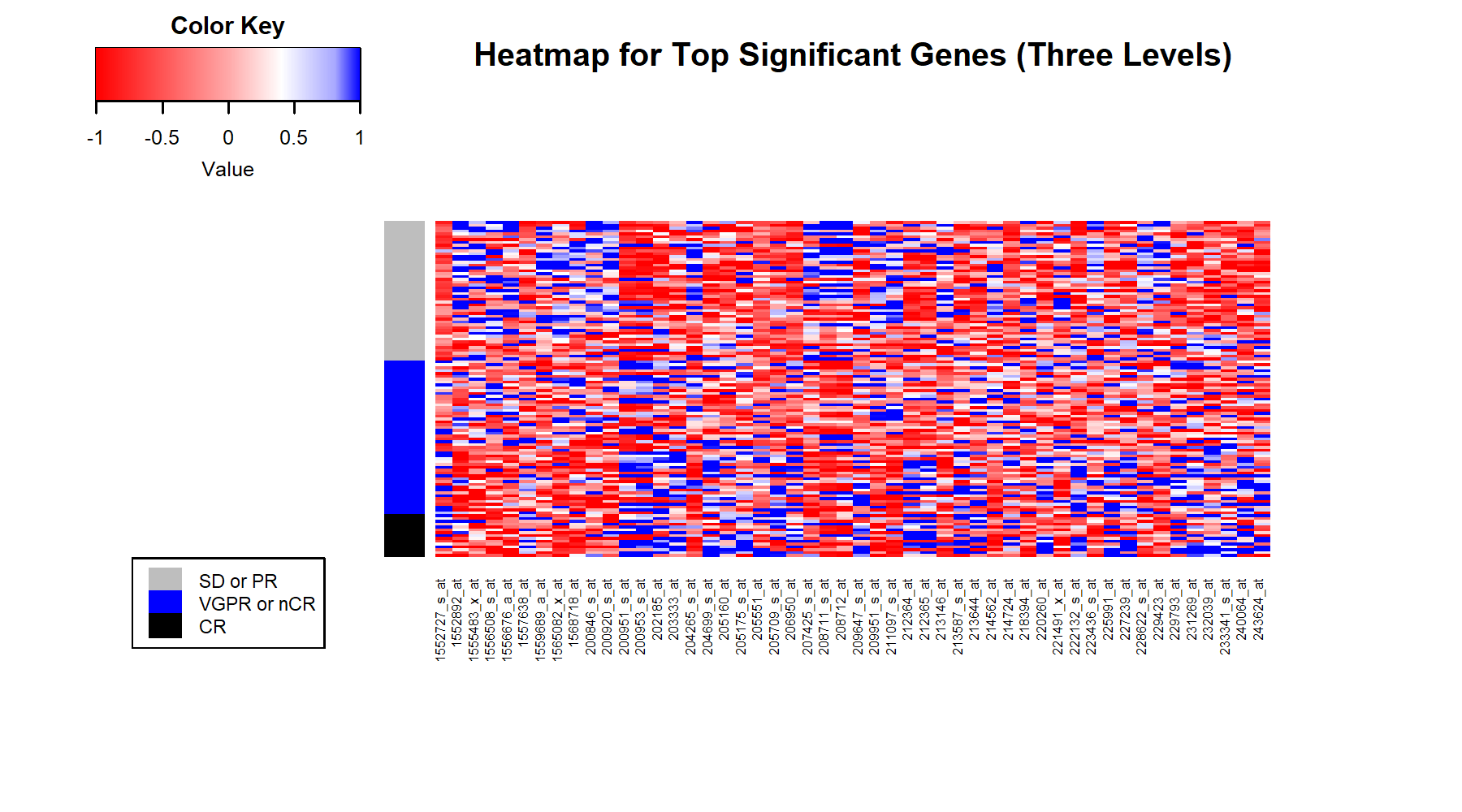


**Figure S2.** Heatmap with Top 50 Significantly Probes with Drug response (Three Levels) in Terragna et al. ([2](#_ENREF_2)).

Figure S.2 is a heatmap for the gene expression of selected top significant 50 probes which were used as predictive genomic factors for the three-level ordinal drug response from Terragna et al. ([2](#_ENREF_2)). The bottom of the heatmap presents the names of the 50 probes; while the left side color bar stands for three-level ordinal drug response, including complete response (CR), very good partial response or near complete response (VGPR or nCR), stable disease or partial response (SD or PR).
